# Diagnosis and Treatment of Pyometra

**Published:** 1909-07-17

**Authors:** 


### Gynæcology.

### DIAGNOSIS AND TREATMENT OF PYOMETRA.

Pyometra, or the accumulation of pus in the uterus, is not common, but when present it causes very unpleasant symptoms, and may be a sign of carcinoma in the lower uterine segment. It is not, however, invariably accompanied by cancerous growths, and its treatment then may be a simple matter.

As a rule the first indication of pyometra is a most offensive purulent vaginal discharge either as a gush or as a slow constant trickle. The offensiveness of the discharge usually calls the patient's attention forcibly to it. In some cases the pus is not discharged, but remains pent up in the uterus; and this is especially the case in cancer of the cervical canal or lower uterine segment. Under these circumstances the presence of a pyometra is not always diagnosed, but is discovered for the first time during the operation for removal of the cancerous uterus.

The pathology of pyometra is interesting, for in none of these cases is there any real obstruction to the outflow of pus; the cervical canal is not actually closed as a rule, but will readily admit a sound. The pus accumulates in the uterus because the uterine muscle degenerates in old age and is replaced largely by fibrous tissue. Thus when pus is secreted by the endometrium, instead of being forced out by uterine contractions, atmospheric pressure and the narrow uterine canal combine to prevent its egress, just as fluid will sometimes remain in an inverted narrow-necked bottle. Then, as more pus accumulates, the fibrous uterine wall distends and dilates, so that even as much as half a pint or more fluid may be accommodated in the uterus. Sooner or later, however, an overflow takes place, either in a gush or in a slow continuous trickle. One is apt to forget that fluids do not readily at any time flow out of the uterus, but must be forced out by uterine contractions. This is the case not only with pus, but also with menstrual secretions and lochial discharges. Practically pyometra can only occur in elderly women whose uterine muscle has degenerated and become incapable of expelling the contents of the organ.

The direct causation of the pus secretion must be infection of the endometrium, in the majority of cases favoured by the presence of a cancerous growth. That it may occur in the absence of a cancer cannot be denied, but the number of recorded cases is limited. Senile endometritis is a condition more often talked of than seen, but it does occur, and very remarkable changes in the endometrium accompany it. As a result of atrophy plus infection the glands of the endometrium disappear, or are destroyed, and the mucosa becomes converted into an inactive form of granulation tissue, secreting pus, but showing very little tendency to heal and cicatrise. The lining of the uterus presents a worm-eaten appearance, brownish in colour, and showing microscopically capillary loops, leucocytic infiltration, and, in fact, the usual appearance of granulation tissue. In cases without cancer there is very little bleeding, only an occasional streak of blood accompanying the purulent discharge. In the presence of a cancer, bleeding is a much more prominent symptom, the pus, as we have already seen, being often pent up in the cavity above the growth.

The presence of pus in the uterus is sometimes accompanied by a marked toxæmia, leading to rises of temperature, drowsiness, and other cerebral symptoms; but generalised septicæmia is almost unknown, because the disease is well localised to the uterus, and the granulation layers form a good protecting barrier against general infection. The diagnosis is sometimes difficult, because there is often a senile vaginitis along with this condition, which by itself may set up a purulent, offensive discharge. Senile vaginitis may exist alone without pyometra, and is usually accompanied by the formation of granulation tissue at the vaginal roof, leading to stenosis of the vagina. Such a condition often gives rise to streaks of blood in the discharge, but is readily distinguished from cancer by passing the finger, when the surfaces will separate, and inspection with a speculum will reveal granulation tissue and not new growth. It must not be forgotten that a new growth may exist above the stenosed portion of the vagina. Senile vaginitis is usually readily cured by antiseptic injections, which, of course, have no influence on pyometra.

The diagnosis of a pyometra may be made quite certain by passing two or three Hegar's dilators; the cervix when thus opened up allows the escape of some at least of the pus from the uterus. If there is a cancerous growth present the dilators can hardly be used without free bleeding, but in the absence of a growth there may be little or no blood. If a growth is suspected, but cannot be seen, the careful use of a curette after dilatation of the cervix will surely bring away some fragments whose nature can be verified without mistake by making microscope sections. There is great danger of perforating the uterus with the curette in these cases, for the walls are often very thin, and are always made more soft than usual. The curette therefore must be used with great caution. In the presence of a cancer naturally the only treatment is removal of the uterus, and the operation of choice will usually be by the abdominal route, for by this means the uterus can be lifted out without soiling the operation field, if Wertheim's method of clamping the vagina and cutting below the clamps is used.

If the vaginal route is chosen the uterus should be washed out first through a small Bozeman's catheter with an efficient antiseptic, and the cervix afterwards closed by transfixing it with a thick piece of silk and tying it tightly round. Where no growth is found, pyometra may be succesfully treated by washing out the cavity with an antiseptic and then draining it by a rubber tube fixed *in situ* by a stitch through the cervix. If this tube is left long enough to protrude from the vulva the uterus may be washed out daily without disturbing the patient. As these patients are often aged this last is not an unimportant consideration.

